# Acquisition of Lower-Limb Motion Characteristics with a Single Inertial Measurement Unit—Validation for Use in Physiotherapy

**DOI:** 10.3390/diagnostics12071640

**Published:** 2022-07-05

**Authors:** Jürgen Mitternacht, Aljoscha Hermann, Patrick Carqueville

**Affiliations:** Sport Equipment and Materials, Technical University of Munich, Boltzmannstraße 15, 85748 Garching, Germany; aljoscha.hermann@tum.de (A.H.); patrick.carqueville@tum.de (P.C.)

**Keywords:** exercise feedback system, IMU validation, knee and ankle rehabilitation, lower-limb-joint kinematics, lower-limb motion analysis, dynamic knee valgus, outcome assessment, physiotherapy

## Abstract

In physiotherapy, there is still a lack of practical measurement options to track the progress of therapy or rehabilitation following injuries to the lower limbs objectively and reproducibly yet simply and with minimal effort and time. We aim at filling this gap with the design of an IMU (inertial measurement unit) system with only one sensor placed on the tibia edge. In our study, the IMU system evaluated a set of 10 motion tests by a score value for each test and stored them in a database for a more reliable longitudinal assessment of the progress. The sensor analyzed the different motion patterns and obtained characteristic physiological parameters, such as angle ranges, and spatial and angular displacements, such as knee valgus under load. The scores represent the patient’s coordination, stability, strength and speed. To validate the IMU system, these scores were compared to corresponding values from a simultaneously recorded marker-based 3D video motion analysis of the measurements from five healthy volunteers. Score differences between the two systems were almost always within 1–3 degrees for angle measurements. Timing-related measurements were nearly completely identical. The tests on the valgus stability of the knee showed equally small deviations but should nevertheless be repeated with patients, because the healthy subjects showed no signs of instability.

## 1. Introduction

The physiotherapeutic treatment of the lower limbs following injury focuses on restoring the normal function of the musculoskeletal system, for example, recovering the range of the angular motion of a joint. Treatment usually requires a long time and patience, as progress in therapy comes gradually. This duration may overextend the consistent long-term assessment performed by the observing therapist and the subjective perception of the patient. A repeated quantitative measurement of the course, on the other hand, can document even small progress with precision over a longer period and can prevent a false subjective opinion about the development; thus, it can have a motivating effect and give the therapist important feedback on the best choice of their methods.

Joint angular motion is the rotation of one body segment against an adjacent one. An example of this is the flexion of the knee joint, which is the rotation of the upper and lower leg with respect to each other. In some joints, angular movements can even be more complex and multiaxial, such as the movement of the foot against the lower leg in the ankle joint, consisting of the upper and lower ankle joints (UAJ, talocrural joint and LAJ, talotarsal joint) [1].

Using an inertial measurement unit (IMU; with a three-axis acceleration sensor and a three-axis gyroscope sensor), the angular orientation and motion of a body segment can be tracked and recorded. The joint angle between two segments could in principle be determined with two such sensors; however, it would take considerable technical and methodical effort to transform the independent local coordinate systems of the two sensors into one another. Performing this requires additional information about the relative orientation, for example, the exact measurement of the direction of the common global external magnetic field. Such a two-sensor system as a feedback control of knee-joint flexion exercises for the patient at home was investigated in [2]. Repeated initial calibration measurements and calculations based on a body segment model for the coordinate fusion transformation were also established in [3]. IMU systems based on this, or on machine learning for motion pattern classification, are widely used in gait analysis, from whole-body measurements with up to 18 sensors, such as in commercial inertial sensor-based motion capture systems Xsens [4] and Noraxon, to dual- or single-sensor systems that analyze only certain aspects of the movement pattern, such as step time and cadence [5] or stride length [6]. In between, there are many other different approaches, for example, in [7], an application for gait classification in patients with different neurological disorders, and in [8], a project with the development goal of a wearable feedback system, both with seven IMU sensors placed on the lower extremities only. Some systems specialize in measuring the movement of only one joint [9]. Using modelling is a bit risky when examining patients, since their motion patterns deviate from the norm, at least at the beginning of therapy. The same would also apply to sensor fusion and Kalman filters, which are mainly used for analyzing cyclical motion patterns with periodically occurring still phases [10]. In any case, the effort would be considerable.

Alternatively, a single sensor may be sufficient, provided that one of the two body segments remains in a clearly defined position and orientation during movement, for example, when the foot stays flat on the ground. Then, it can be assumed that a one-sensor system may be even more accurate than a two-sensor system if the design of the tests is chosen appropriately.

Such a single-sensor system was investigated in this study. It consists of an IMU sensor that has onboard pre-installed algorithms for detecting and measuring different movement patterns. Depending on the current status of the physiotherapy treatment, a suitable selection can be made from a set of 10 tests. The current progress can be assessed by comparison with previous control measurements, or, in the case of one-sided injury, by the comparison of the right side with the left side.

The sensor technology used is, of course, important, but the choice of a suitable method from a number of different approaches to evaluate the measurements is also crucial for success. An analysis of therapeutic exercises using a single IMU sensor was previously described in [11], which demonstrated the verifiability of the quality of motion execution with the help of purely statistical analyses of the maxima and minima of the measured acceleration and angular velocity curves. Other authors have analyzed the Fourier coefficients of the angular velocities of cyclically repeated motion of the hip and knee joints measured with an IMU sensor as input variable to a segment chain model of the leg [12].

The examined IMU system primarily derives “classical” physiological parameters analytically from the measurements that the therapist would otherwise only assess visually and subjectively. These are mainly linear and angular ranges of motion (ROMs), tilt angles and timing and counting tasks that the system renders at the end of each test in the form of a single numerical score value.

An overview of the current status of IMU-based measurement methods for the kinematics of the lower extremities was provided by [13].

The core focus of this study was the validation of the aforementioned single-sensor IMU system and the 10 different motion tests that can be carried out with it. The study primarily addresses therapists and physicians who want to have a validated, easy-to-use and cost-effective system in their practice such as the one presented in order to quantify and document their therapy courses with descriptive and objective measured values. With the help of this study, they should be able to better estimate the potential and reliability, but also the limitations, of the system.

The developers of similar products should also feel addressed. Explanatory notes and critiques about the measurement technique details, basic remarks on the choice or definition of the analyzed parameters and the underlying algorithms are primarily aimed at them.

## 2. Materials and Methods

### 2.1. The IMU System

In the presented study, an Orthelligent system from OPED™ was examined, which uses an IMU sensor TDK InvenSense ICM-20948, 9-axis, 200 Hz MEMS MotionTracking™ device. The sensor must be attached securely to the relevant body segment, so that accelerations and rotations are completely transferred. The bony edge of the tibia is the preferred location to attach the sensor as the tibia lies just below the surface of the skin, with few muscles or other soft-tissue structures in between that could mechanically isolate the motion of the IMU sensor from the tibia motion. This was also identified as the preferred position for the sensor by [10].

**Figure 1:**
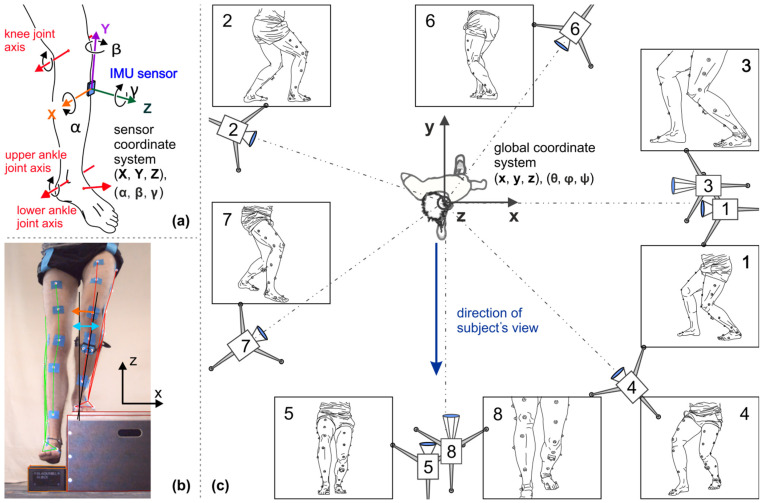
(**a**) Axis systems of the lower leg and IMU sensor. (**b**) Video image from camera 5: light-reflecting markers in extended standard marker setup for motion Test 6 One-Leg Squat. (**c**) Arrangement of the 8 cameras of the video-MA: top view, not exactly scaled; zoom cameras 3 and 8 for close-up; camera images show Test 2 Ankle dorsiflexion.

The sensor has to be attached and aligned in such a way that its axis system is oriented along the anatomical axes, i.e., with the *Y*-axis parallel to the longitudinal axis of the tibia and the *Z*-axis corresponding to the sagittal tibia edge orientation (see Figure 1a). When there is multidimensional motion, this enables the subordinate motion and angle components to be differentiated and analyzed separately from the dominant motion components.

In order to enable the conversion of the angular movements of the tibia into linear displacements during some of the tests, the position of the sensor at the tibia edge was chosen as 38 cm above the ground when standing upright during the study.

The same micro-chip, ICM-20948, was used by the authors of [14] for a surface electromyography (sEMG) armband. They described in more technical detail the electronic components for embedding this sensor in a wearable measuring system.

By using wearable micro-sensors, the measurement potential in biomechanics can be widely increased, but additional problems occur. Signal drift can be observed with IMU sensors when a motion variable is determined by integrating a measurement parameter with an unknown or nonspecific floating signal offset. Such variables are velocity v, determined by integrating acceleration a; position x, determined by integrating v (i.e., double integration of acceleration a); and the angle, determined by integrating the angular velocity. The examined system tries in these cases to avoid drift errors by dividing longer analysis sequences in sufficiently short sections. However, the width of the time slice must be determined in such a way that the result is negligibly affected.

The angular position of the sensor at rest can be determined from the distribution of gravity acceleration g in the three components of the measured acceleration, a = (a_x_, a_y_, a_z_).

### 2.2. Video Motion Analysis

Within this study, a marker-based 3D video motion analysis (video-MA or VMA; Figure 1c) served as a reference to validate the IMU measuring system, a SIMI^®^ system, software version 9.22, with 8 time-synchronous dual LAN HD CCD cameras with a frame rate of 50 images per second. Four cameras were positioned perpendicular to the main planes of motion, xz (frontal plane) and yz (sagittal plane); two of them had zoom lenses. This method additionally enabled 2D motion analyses in relation to these planes of the global coordinate system. A common method for measuring the dynamic knee valgus as the frontal plane projection angle (FPPA) during single-leg squatting and drop jump has been previously described [15,16].

The measurements of the IMU sensor were compared with those of the video-MA for validation. However, the reference frames differed, with the video-MA measuring in the global coordinate system and the IMU sensor in its own local system. Where necessary, the data from the video-MA were transformed into the local tibia coordinate system, which corresponds to the sensor coordinate system. Then, all parameters retained a direct reference to the physiological structures. However, this transformation could not be implemented with equal accuracy for all examined motion patterns.

With the video-MA, angles, of course, could have also been determined in the same way adopted by the IMU system, i.e., the measurement and calculation of the tibial angular motion solely determined from the direction vector of the tibia edge to a neighboring segment assumed to be fixed. However, that would have not been appropriate, since possible system-related errors of the one-sensor IMU system would have been repeated and could no longer have been recognized when the systems’ results were to be compared. Instead, the joint angles of the video-MA were always determined classically from the coordinates of the two body segments involved.

Light-reflecting markers were also attached to the IMU sensor in order to detect any movements of the sensor relative to the tibia. However, these were not used for any other motion analyses, but instead, the marker just above the sensor on the tibia head was.

The IMU system calculates a single angle or motion score value from its measurements. The respective score definitions of the IMU system were adopted by the video-MA; they may differ from other common definitions or from comparable measurement systems.

### 2.3. Volunteers

Five volunteers took part in the study. They were informed in detail, and they signed a declaration of consent. The research project was carried out in accordance with the ethical standards of the Declaration of Helsinki.

The subjects were male, healthy, without current or recent injuries and between 25 and 30 (with one being aged 61 years old). Their height ranged from 171 cm to 180 cm and their weight from 60 kg to 76 kg. The tests were carried out barefoot, in the same order (Tests 1–10) for all test subjects on a concrete floor with a plastic covering and an industrial carpet. None of the tests required high performance from the subjects.

Due to the large number of individual tests including repetitions, the number of subjects was kept low (N = 5). The long time required for the study resulted solely from the effort required for the preparation and evaluation of the video-MA. The application and measurement time of the IMU system alone, in comparison, was completely negligible. A second reason for the narrow limitation of the number of subjects was the assumption from the beginning that healthy subjects might not be sufficient for a clear statement in all tests.

### 2.4. The Motion Tests

**Figure 2:**
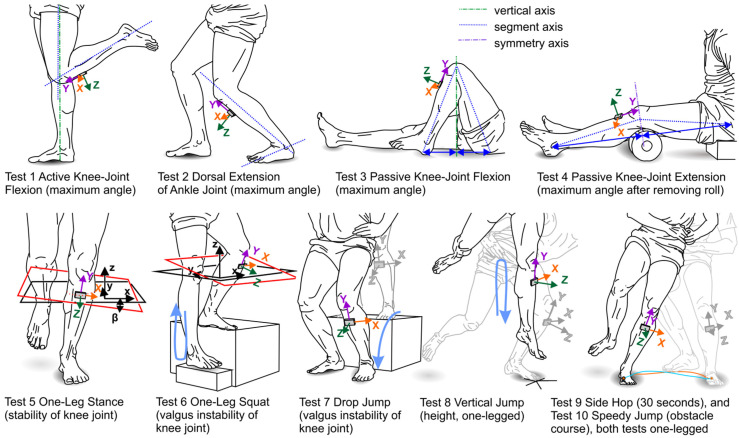
Motion patterns of the 10 tests provided by the IMU measurement system and examined in this study.

A set of 10 motion tests (see Figure 2) was defined for the application of the IMU system; these tests demonstrate the motor skills that are addressed in physiotherapy after knee and ankle injuries. The tests can be assigned to 3 categories that test the joint mobility, coordination, power and speed in relation to the physiological or physiotherapeutic goals relevant to the injury and the current state of treatment progress.

The 10 tests also present very different requirements in terms of the evaluation of the measurement data. In the subsections below, they are separated into 3 groups, which, to a certain extent, relate to each other in terms of the evaluation technique and the character of the motion. Set A tests are measurements of an angular ROM, while Set B tests measure the variance of a motion component with a small amplitude or a small component of an angle in a more complex motion pattern, and Set C tests are jump tests in which jump-off and landing events are recognized and time spans are determined.

The tests correspond to common reliable functional performance tests for the lower extremities [17].

#### 2.4.1. Set A, Tests 1–4—Measurement of Angular ROM

In four tests, the active or passive ROM of the knee or ankle joint was examined. The motion starts from a non-moving initial position, is rather slow and ends at a non-moving position again.

**Test 1 Active Knee-Joint Flexion** measured the maximum active knee-joint flexion angle while standing. The subject stood on one leg and then bent the knee joint of the other leg. The thigh had to maintain its vertical orientation. When undertaken in a clinical situation, patients can perform the motion using an object (e.g., table leg) to prevent the ante-flexion of the thigh in the hip joint. The test was repeated 5 times—thrice with maximum possible knee flection and twice with reduced knee flexion. The angular position of the tibia edge in the starting position and the maximum change in angle relative to that were determined.

**Test 2 Dorsal Extension of Ankle Joint** measured the passive dorsal extension ROM of the ankle while standing. The dorsal extension in the upper ankle joint is the forward tilt of the lower leg against the foot resting flat on the floor. To measure this, the subjects were required to stand with their feet in step position. The angle was measured in the recessed ankle joint. This test was repeated 5 times—twice with a reduced range of motion. Within this test the heel of the leg that was being tested was not allowed to rise significantly, as this would have caused the sensor to overestimate the actual dorsal extension angle. In order to identify possible errors of the IMU system, the video-MA additionally determined the angular movements of the foot relative to the ground.

**Test 3 Passive Knee-Joint Flexion** measured the knee flexion deficit passively while sitting. The flexion angle of the knee joint comprises the angular motion of the lower leg and the thigh. To measure this, the subject was requested to sit on the floor with his leg outstretched and then to slowly pull the leg into the flexion position, pulling the thigh with his hands if necessary. The IMU sensor measured the angular motion of the tibia axis, starting from the maximum outstretched position.

The measurements of the video-MA demonstrated that the angular motions of the two segments were the same, but with opposite sign. Therefore, the lower leg is functionally just as long as the thigh is, which has also been identified in other studies [18,19]. The IMU system assumed equality; thereby, it assumed the ROM of the knee-joint flexion to be exactly double the measured ROM of the tibia flexion.

**Test 4 Passive Knee-Joint Extension** measured the extension deficit of the knee, passively moved while sitting. For this test, each subject was required to sit on a box 12 cm high with his legs outstretched. At the start of the test, a roller was placed under the knee joint, supporting it in a defined initial flexion position. The roller was then removed, causing the knee to be passively stretched by the weight of the leg. The IMU sensor measured this change in the angle of the tibia edge, which was reduced in the case of an extension deficit. This change in angle was output, not the absolute angular position of the knee joint after extension. As in Test 3, the knee-joint angle ROM was assumed to be double the amount of the measured tibia edge angle ROM.

#### 2.4.2. Set B, Tests 5–7—Stability and Knee Valgus

These tests evaluated the mediolateral movement of the knee, the dynamic knee valgus, under different physiological requirements and loads. The parameter analyzed in each tests quantifies instability or weakness due to an injury, which should diminish with recovery in the course of therapy and should be close to zero in healthy individuals. The tests were, therefore, more demanding in terms of resolution than the tests of Set A. The analysis was made even more difficult by the greater complexity of the motion patterns. From Tests 5 to 7, the motion speed increased considerably and the actual measurement time reduced from 20 s to 0.1 s.

A literature review on knee valgus instability, injury prevention and the specificity of single-leg movement tasks (such as Tests 5 and 6) in comparison to double-leg tasks (Test 7) can be found in [20]. The diagnostic significance of the single-leg balance (SLB) test in chronic ankle instability was described in [21]. In a prospective cohort study [22], it was demonstrated that there is an association between a positive SLB test and the risk of ankle sprains.

**Test 5 One-Leg Stance** measured a (small) motion component, the mediolateral movements of the knee required to maintain balance and their variance over 20 s. The test describes the motor balance control of the patient. The forces and moments involved must be transmitted by the muscles that move and stabilize the knee joint. The subjects were required to stand on one leg with the knee joint slightly bent (≈10 degrees). The test was repeated 3 times by each volunteer.

From the angular motion of the tibia, the IMU system calculated the linear motion of the sensor in the horizontal plane at an assumed height of the sensor of 38 cm above the ground when standing upright. The IMU sensor assumed that the base of the tibia axis was fixed on the ground. Only the mediolateral motion component in the X-direction of the sensor system was considered.

An evaluation window of 200 milliseconds in duration was run over the data. It included the latest 40 currently measured values. The current ROM_i_ was determined in this window (maximum X-position minus minimum X-position = ∆X_i_, where i = from 1 to 100 within 20 s). Finally, the geometrical mean, ∆X, was calculated from the sum of all ∆X_i_. An evaluation of the entire 20 s of measurement time at once, e.g., by calculating the mean value and standard deviation, could possibly be overlaid by a sensor drift or a slow cumulative change in the subject’s posture, and it would need 100 times the data stored on the sensor.

The video-MA emulated this procedure with its data. The amplitude of motion, ∆x_i_, in each time window and the tilt angle of the tibia axis, ∆φ, were determined directly from the measured 3D marker coordinates. The 200-millisecond time window corresponded to 11 video images.

The other two tests of Set B were about the valgus instability of the knee joint. They also measured a small sub-component of an angle parameter, the mediolateral tilt angle of the tibia, but with a larger simultaneous ROM of the main component, the anteflexion of the tibia.

During **Test 6 One-Leg Squat**, the subjects had to stand on the edge of a 32 cm high box. They had to bend the stance leg until the heel of the contralateral leg touched a second box and finally outstretch again. The height of the second box (12 cm) determined the depth of the squat (20 cm). In comparison to a one-legged squat on the floor, the position and motion of the contralateral leg and the depth of the squat were clearly defined.

The tibia axis obtained maximum tilt in the medial direction when the motion reversed from the deepest flexion at the beginning of the knee-joint re-extension. The sensor’s analysis started at that moment in the motion sequence when the lower leg’s anteflexion (forward tilt) exceeded 10 degrees compared with the starting position. The analysis ended when the forward tilt angle fell below 3 degrees again. In this test, the size of the medial tilt angle based on the initial orientation was recognized as a criterion for the valgus instability of the knee joint. During the lateromedial tilt of the tibia, there was also a significant ante-flexion due to the knee flexion.

In **Test 7 Drop Jump**, the subjects were to jump two-legged off a box and land two-legged on the floor. From the landing, they had to jump as smoothly as possible, without interruption, two-legged up on the spot again. The IMU system measured the linear medial shift, ∆x, of the knee joint during the first landing in this test by the double integration of the mediolateral acceleration. The angle, ∆φ, of the tibia tilting towards the medial direction was then calculated and therapeutically evaluated as the corresponding residual valgus instability of the knee joint.

The maximum impact of the sensor’s vertical acceleration and the first mediolateral acceleration oscillation determine the landing after the jump. Two zero crossings of the transverse acceleration, a_x_(t_0_) = 0 and a_x_(t_1_) = 0, define range ∆t = t_1_ − t_0_ for the double integration to medial shift ∆x. Both a possible initial horizontal speed v_0_ = v_x_(t_0_) of the sensor relative to the global coordinate system at the first ground contact and initial position x_0_ are unknown to the sensor and are set to 0. The transverse acceleration, a_x_, is integrated over ∆t to transverse speed v_1_ = v_x_(t_1_) and then integrated again to transverse position x_1_ = x(t_1_), i.e., medial shift ∆x. The velocity, v_1_, at t_1_ is the maximum of the medially directed speed component. The knee continues to move in the same direction after t_1_ but now decelerates until the maximum medial shift is reached. This is more than twice as large as ∆x and has to be taken into account when comparing the test result, for example, with maximum amplitude ∆x determined from a frontal video recording.

Again, both parameters ∆x and ∆φ were measured directly by the video-MA. However, because of the lower time resolution of the video-MA and the very short analysis time interval ∆t = t_1_ − t_0_ ≤ 0.1 s of the IMU sensor, for the video-MA, this was a maximum of only 6 measured values. As a result of the limited ability to exactly synchronize the two measurement systems, inaccuracies could not be fully avoided.

#### 2.4.3. Set C, Tests 8–10—Jump Tests

There were 3 jump tests, all of which were based on the accurate detection of the ground contact moments of jump-off and landing and the time interval in between.

**Test 8 Vertical Jump** measured height h of a one-legged jump. Each subject was requested to jump 3 times with maximum and reduced jump height. The IMU system determined the jump height assuming a parabolic flight with flight time ∆t:

(1) $$h=\frac{1}{2}g{\left(\frac{∆t}{2}\right)}^{2}$$

where g is gravity acceleration (9.81 m/s^2^).

The video-MA derived the jump height directly from the vertical z-position of several markers on the lower leg and thigh—maximum position z minus position z_0_ when standing upright before the jump. The different methods to determine the jump height were described and compared by [23]. In addition, height was calculated from flight time, but the IMU sensor attached to the back of the foot [24] also found accurate results.

**Test 9 Side Hop** counted the number of one-legged jumps. Within this test, the subjects were instructed to jump on one leg laterally and medially between two marks on the floor as often as possible within 30 s. The first take-off started the measurement. The IMU sensor counted the number of valid jumps and should have recognized and ignored those which were invalid (intermediate jumps on the spot). A correlation between ankle instability and poor results has been identified in the side-hop test [25,26].

**Test 10 Speedy Jump** tested the subject while he quickly jumped on one leg over low foam obstacles arranged in a zigzag course. The IMU sensor started timing with the first jump-off and stopped with the last landing. Intermediate jumps were accepted here. When undertaking this in clinical practice the therapist should check that the order of the jumps is executed correctly.

## 3. Results

### 3.1. Overview

**Figure 3:**
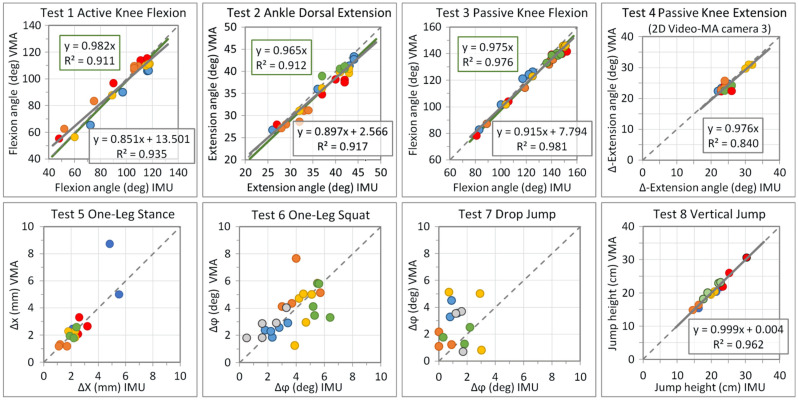
Comparison of the measurement results between IMU sensor and video-MA. The control points represent the measurements with both systems and lie exactly on the diagonal if the score values of the IMU sensor and the video-MA are perfectly matched, while different colors represent multiple measurement points of the test persons.

The score values of the IMU system and the 3D video-MA obtained in the tests were compared with each other using scatter plots, thereby allowing us to define the position of an associated control point (Figure 3). Ideally, if the results of both systems are the same, the control point lies exactly on the diagonal of the angle_VMA_-versus-angle_IMU_ diagram or x_VMA_-versus-x_IMU_ diagram. For tests in which the motion amplitude, e.g., the maximum knee flexion, could be varied intentionally, the trend line and coefficient of determination R^2^ were calculated and are shown in the diagram, although the measured values were, of course, not statistically completely evenly distributed. In all tests, the RMS values (root mean square) of the differences between the score values of the IMU system and video-MA were the criteria for evaluating the IMU system (Table 1, highlighted columns). Unequal values of the standard deviation, SD, compared with the RMS indicated a systematic deviation of the score values between the IMU system and the video-MA; otherwise, the differences were more or less randomly distributed due to the measurement inaccuracies of one or both measuring systems.

### 3.2. Set A Tests—Measurement of Angular ROM

Tests 1–4 measured the angular ROMs of the knee and ankle. The angle differences between the IMU system and video-MA were 1–2 degrees in Test 2 (passive ankle dorsiflexion) and Test 4 (passive knee extension), 4 degrees in Test 3 (passive knee flexion while sitting) and 6 degrees in Test 1 (active knee flexion while standing). The SD-referred relative differences in the two measurement systems lay between 3% and 6% and between 3.5% and 7.5% for the RMS-referred differences, which included systematic deviations. A certain but subordinate part of these differences seemed to be systematically test-related, which a more detailed examination of the diagrams also suggested, as explained below.

In **Test 1 Active Knee-Joint Flexion**, not all subjects held the thigh exactly vertical, but slightly flexed forward in the hip joint during knee flexion. This added to the real total knee-joint angle but could not be detected by the IMU sensor. In contrast, the orientation of the thigh was geometrically clearly defined in **Test 3 Passive Knee-Joint Flexion**.

With the largest flexion values in Test 1 and Test 3, there was a group of control points in the associated diagrams that deviated to slightly lower angle values of the video-MA. The colliding soft tissue of the lower leg and thigh reduced any further motion of the light-reflecting skin markers.

In **Test 2 Dorsal Extension of the Ankle Joint**, almost all control points were shifted a few degrees to the right of the diagonal, producing score values approximately 1–4 degrees higher for the IMU system when compared with the video-MA. The sensor could not detect the angular tilting of the foot towards the ground. With the forward tilt of the tibia, the COP shifted towards the forefoot and relieved the heel accordingly, although it did not lose contact with the ground. Correspondingly, the IMU system overestimated the score values, with angle values on average 1.3 degrees larger than those of the video-MA.

In **Test 4 Passive Knee-Joint Extension**, the absolute differences of the measured values between the IMU sensor and video-MA were even smaller than in Test 3. This was on average approximately 1.3 degrees for both 2D data and 3D data when projected on the plane of motion. The percentage differences were slightly higher than in Tests 1–3 because of the smaller angular ROM. Most of the control points were close together, between approximately 22 and 26 degrees, with one subject having 31 ± 1 degrees. None of the subjects had any extension deficit; the one subject differing even had a few degrees of hyperextension. However, this parameter is only very conditionally suitable for comparisons between different subjects, as the starting angle can be slightly different depending on the anatomical constitution, for example, the leg length.

### 3.3. Set B Tests—Knee Valgus Instability

Tests 5–7 measured the mediolateral displacement of the tibia head (or sensor) or the mediolateral tilt angle of the tibia.

**Test 5 One-Leg Stance:** In almost all individual tests, the motion ∆X (and ∆x) averaged over the 20-s test time was only a few millimeters, with an average of ∆X = 2.5 mm for the IMU sensor and ∆x = 2.7 mm for the video-MA. The RMS-averaged difference between the two systems was 1.1 mm.

The data of the video-MA can be used to demonstrate the importance of the chosen sliding analysis window width on the result. The variation in the width shows an approximately logarithmically increasing value to the resulting ∆x when increasing the window width from 0.2 to 2.0 s. The reasons for this were low-frequency motion components down to 1 Hz and lower, which showed up in a frequency analysis. They had greater amplitude and power than higher frequency components, which were probably dampened by the mass inertia of the body segments.

With an increase in the window width from 0.2 s to 0.5 s, ∆x increased by an average of 71.5 ± 7.5 percent across all subjects and individual tests, and with an enlargement of the window width to 1.0 s, ∆x increased by 134.3 ± 14.0 percent. However, the ranking order of all test results and the relative proportions were always retained. Comparing the data to other measurement systems, this would essentially amount to a simple rescaling of the numerical values.

The same applies when comparing with the width of the standard distribution of all 1000 measured values for position x of the tibia head during the 20-s measurement period. This was 3–4 times greater than the value based on the 0.2-s sliding analysis window for all tests performed. However, these differences were not a fault but reflect different possible approaches to defining parameters.

Independent of the selected analysis window width, the results of stability Test 5 could not be directly compared with those of stabilometry-measuring systems. The 2D trajectories of the IMU sensor or the tibia plateau marker looked similar to the graphical representations of those systems but fundamentally differed from static and dynamic posturography on a fixed or movable standing platform. The latter usually has an integrated measurement of the force application point’s motion on the ground (center of pressure, COP). For more information, see [27,28].

**Test 6 One-Leg Squat and Test 7 Drop Jump:** As in the previous test, Test 5 One-Leg Stance, the therapeutic goal is to minimize the valgus instability of the knee, which shows in a medial shift, ∆x, and in a medial tilt, ∆φ, under load. The score values determined with both measurement systems for **Test 6 One-Leg Squat** were between about 2 and 6 degrees, on average 3.8 ± 1.6 degrees. The individual standard deviations of each of the five test repetitions were closer together, between 1 and 2 degrees, shown as the color-coded control point groups in the diagram in Figure 3 (Test 6). This suggests that the inter-individual differences have a real basis in a slightly different movement pattern. This could be, for example, the rotational movement of the foot with respect to its longitudinal axis (see Section 3.5 about the mechanical isolation of tibia tilt and tibia shift) or a real difference in knee stability. Higher mean values of the medial shift, 8 ± 6 degrees and 6 ± 9 degrees in patients and test persons, respectively, were reported by [15] in their 2D-FPPA study.

The RMS mean difference between the score values of the measuring systems was 1.4 degrees, with a minimum value of -3.7 degrees and a maximum value of 3.1 degrees. That is about half the range of the measured score values; therefore, the percentage deviation in Table 1 was RMS (%) = 54%. However, the healthy subjects in the study did not show any knee instability, so the score values were expected to be close to zero.

**Figure 4:**
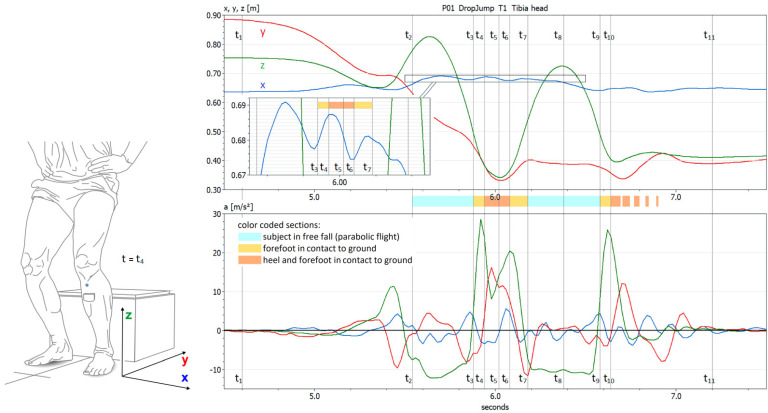
Test 7 Drop Jump. Focus of the measurement: the mediolateral movement of the tibia head (blue curves). Upper diagram: 3D coordinates (x, y, z) of the tibia head marker measured by the video-MA. Lower diagram: acceleration (a_x_, a_y_, a_z_) of the tibia head marker determined by double derivation of the coordinates. Events t_i_ are characteristic moments of the motion pattern such as forefoot or heel touch down. In the example shown, the tibia head shifts during landing between t_4_ and t_6_ (the foot lies flat on the ground) by 13 mm towards the medial.

**Test 7 Drop Jump** consisted of a two-legged jump from a box. Figure 4 shows an example of a measurement of the video-MA. In the upper diagram, the 3D coordinates of the tibia plateau marker are shown over 3 s, while in the lower diagram, the acceleration values were derived from the coordinates by double differentiation. The IMU sensor went the opposite way; it measured the acceleration and calculated the coordinates by double integration. The relevant parameter was the mediolateral motion, in both diagrams represented by the blue curve.

The tests with the subjects did not show any correlations between the score values of the two measurement systems. A number of reasons may be responsible for this.

The mean of the score values, ∆φ, was only 1.3 degrees for the IMU system and 2.6 degrees for the video-MA, which corresponded to medial shifts of ∆X = 8.6 mm and ∆x = 16.2 mm, respectively, directly measured by the video-MA. As in the previous test, the reason for this may be that the healthy subjects in the study had no visible signs or known symptoms of knee valgus instability.

The variations in mediolateral component x were by far the smallest over the entire duration of the test. Because the subject crouches when landing, there is simultaneously much greater tibia ante-flexion to the medial tilting of the tibia. One cannot rule out some crosstalk with both measurement systems from the flexion component to the mediolateral component. The same applies to the acceleration components that the IMU sensor measured.

The IMU system determined ∆φ by the double integration of transverse acceleration a_x_ and a, following trigonometric conversion. This presupposes that the vertex of angle φ is at ground level. As mentioned for Test 5 and Test 6, a rotation of the foot around its longitudinal axis, which in this test presents as an even more large-scale and complex change from supination and plantarflexion towards pronation and dorsal extension during landing, can decouple the measurement curves for x and φ (see Section 3.5 about the mechanical isolation of tibia tilt and tibia shift).

The IMU system cannot detect a horizontal speed component v_x_ ≠ 0 before landing; therefore, it may integrate parts of braking–acceleration shock a_x_ during landing with the medial shift. In principle, it can be permissible to set v_0_ and x_0_ to 0, since the measured values take on the character of sensor or tibial internal local parameters. However, an initial horizontal velocity v_0_ ≠ 0 must result in an acceleration shock when the foot’s v_x_ is stopped in the moment of contact with the ground.

(In addition, this may lead to contrary interpretations. For example, the sensor perceives an acceleration in the medial direction, assumes v_0_ = 0.0 and calculates a shift ∆x to medial, while the observer in the outer coordinate system sees an existing movement with v_0_ > 0.0 laterally, which is stopped by the medial acceleration. During the same period of time, the sensor still moves laterally for the observer, who sees a shift ∆x to lateral. Both interpretations are not wrong in themselves.)

With the knee instability of patients, however, their pathological medial shift may probably dominate and be unambiguous. Another question then arises as to whether the integration time of 0.1 s is sufficient to fully capture a medial movement with larger amplitude.

### 3.4. Set C Tests—Jump Tests

In the score value diagram (Figure 3) for **Test 8 Vertical Jump**, the control points of the jump heights are almost perfectly on the diagonal. The RMS mean difference between the two measuring systems was 0.8 cm, corresponding to 3.8%. In **Test 9 Side Hop**, the subjects achieved between 28 and 64 jumps within the 30 s of test time, in accordance with the video recording. In **Test 10 Speedy Jump**, the subjects needed between 6.5 and 9.5 s for the course. The pairs of measured values differed by a maximum of 0.2 s.

### 3.5. Decoupling (Mechanical Isolation) of Tibia Tilt and Tibia Shift

When standing on one leg, the force application point (center of pressure, COP) must be permanently shifted around within the contact surface via muscular torques for corrections to keep balance. This results in roll and pitch movements of the foot (more precisely, primarily the bony structures within the soft-tissue pads move). Because of these angular movements of the foot skeleton, the talus and tibia are also slightly shifted around in the horizontal plane, but with only low amplitude.

**Figure 5:**
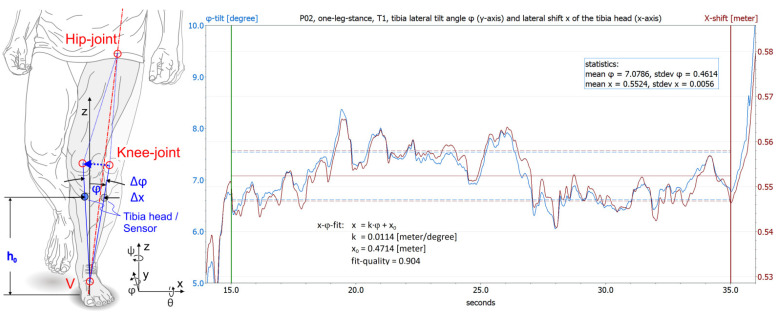
Left schematic: Mediolateral tilt ∆φ (13 degrees) and shift ∆x (8.5 cm) under load in One-Leg Stance (Test 5) of a hypothetical patient with knee valgus instability. Red dotted line: swivel axis through hip joint and ankle joint; vertex V on the ground. Diagram: Measured video-MA data of one of the healthy volunteers, with mediolateral tilt ∆φ (0.92 degrees) of the tibia edge (blue curve, left scale) and shift ∆x (1.1 cm) of the tibia head (red curve, right scale). Test duration, 20 s. The right scale’s range is set in such a way that the two measurement curves fit together optimally. Fit quality equals the square of the correlation coefficient.

The diagram in Figure 5 shows an example from Test 5 One-Leg Stance, which is supposed to represent this problem for Tests 5–7 most clearly because of its long test time. The diagram shows a comparison of tibia tilt angle φ (the mediolateral tilt angle of the tibia) and tibia shift x (the mediolateral displacement of the tibia head), both measured with the video-MA. The right scale’s range of the diagram is set in such a way that the two measurement curves fit together with minimal RMS error (a passive coordinate transformation without changing the measured variables). For this purpose, scaling factor k of linear fit function x = k·φ + x_0_ was calculated.

Assuming that tilt pivot point V is at ground level (Figure 5, left), tilt angle range ∆φ and lateral shift ∆x can be converted into each other:

h = h_0_∙cos(φ) (2)

x = h_0_∙sin(φ) (3)

For values φ < 15 degrees, the following applies in good proximity (error ≤ 1%): sin(φ) ≅ φ (in radians). Thus:

∆x = x_2_ − x_1_ = h_0_∙(φ_2_ − φ_1_) = h_0_∙∆φ (4)

It turns out that in all subjects, the trigonometric relationship (4) can only be fulfilled with the measured values of φ and x if h is significantly greater than the height h_0_ of the tibia head above the ground. Individually different, the values of h are between 25% and 90% greater than h_0_. That means, in Tests 5–7, there may be movements of the foot which displace the lower leg mediolateral without the same corresponding change in the mediolateral tilt angle.

In addition, the axis of the lower ankle joint (subtalar joint, consisting of the posterior talocalcaneal joint and anterior acetabulum pedis) deviates significantly from the longitudinal axis of the foot towards the front medial and upwards. A rotation of the foot around its longitudinal axis, therefore, always induces a rotation of the tibia around its axis [29]. In other words [1], anatomically and biomechanically, this process represents conjoined, synchronous motion within the three mobile segments of the hindfoot: the (upper) ankle joint, the posterior subtalar joint and the anterior subtalar joint. Other authors quantified this mechanical transfer in vitro and found an average of 74% tibia external rotation in calcaneal foot inversion and 46% transfer to tibia internal rotation in eversion [30,31].

## 4. Discussion

Although this was a study with only a few volunteers, the results suggest that an IMU measurement system that only uses a single sensor fixed to the tibia is suitable for the objective rating of characteristic motion parameters of the lower limb. The investigated IMU system was designed for use in rehabilitation and physiotherapy practice for reliable longitudinal examinations on treatment progress. It outputs a score value for each of 10 different implemented motion tests, based on angular ranges, displacements or counting and timing values.

The precise and free-of-play attachment of the sensor is crucial, especially the exact frontal alignment to the tibia edge. The adjacent body segment relative to which angular motion is determined must maintain a fixed or known position and orientation during the observed motion sequences.

The golden standard for validation was a 3D video motion analysis system. The intrinsic accuracy of angle measurements turned out to be from 1 to a maximum of 2 degrees for both IMU system and video-MA. The mean RMS-averaged differences between the systems were 2 degrees or less in all tests that measured an angle, with certain exceptions.

Slightly larger deviations occurred with the largest flexion angles of the knee when the soft-tissue shifts of the skin markers reduced the results of the video-MA by a few degrees. Therefore, in Test 3 (Passive Knee-Joint Flexion), the difference was 4.5 degrees.

The IMU system lost accuracy if the adjacent body segment (thigh or foot, depending on the test) was not in a still position. In Test 1 (Active Knee-Joint Flexion), the RMS-averaged difference was 6.1 degrees, the maximum discrepancy among all tests. The subjects were instructed to perform freestanding without mechanical abutment against thigh anteflexion, which severely restricted video-MA recording. In therapeutic practice, this source of error could, therefore, be largely avoided.

The corrective movements of the foot when maintaining balance in a single-leg stance may displace the lower leg mediolateral without the same corresponding change in the mediolateral tilt angle in Test 5 One-Leg Stance and Test 6 One-Leg Squat, two tests to quantify ankle or knee instability. Therefore, the tilt angle and the segment’s shift are partially mechanically isolated (decoupled) and can no longer be easily converted into each other. These shifting displacements are physiologically limited in their range and thus may play a smaller proportional role in patients with knee-joint instabilities and higher knee valgus under load than in the healthy volunteers in our study.

In double-legged drop jumps (Test 7), the movements of the foot are more significant. During landing, both feet tilt from an initial plantar flexion and supination position to dorsal extension and pronation in the upper and lower ankle joints, superimposed by some longitudinal rotation of the tibia [30,31]. The medial tilt of the tibia probably only takes place afterwards, when the foot is already flat on the ground and the braking acceleration becomes a maximum. However, this could also somewhat overlap in terms of time.

In Test 6 One-Leg Squat and Test 7 Drop Jump, the measured values were rather close to both systems’ ranges of accuracy. None of the subjects had any knee-joint instability; therefore, minimal score values were to be expected. Therefore, it is necessary to repeat these tests on patients to reliably prove the measurability of a medial shift of the knee joint. The ability to easily, objectively and precisely measure this valgus instability would be of particular value. The biomechanical and epidemiological relationships between knee valgus instability and ACL (anterior cruciate ligament) injuries were described by [32]. The residual instability of the knee joint after ACL replacement is a sensitive predictor for a second injury of the anterior ligament when resuming sporting activities [33]. The significance of these tests is high in the context of the risk and possible prevention of knee and ACL injuries [34,35]. However, [36] found very different valgus tilt angles of the knee joint in a 3D video-MA, between 1.9 ± 4.3 and 10.3 ± 3.4 degrees in a drop jump test, even in non-injured elite soccer players.

The comparison of the valgus angles in the one-leg squat versus drop jump in Table 1 (3.78 degrees as calculated by the IMU system and 3.77 degrees as calculated by the video-MA versus 1.29 degrees and 2.57 degrees, respectively) suggests a higher sensitivity of the one-leg squat test (despite all the necessary restraint due to the small number of subjects). Differences in the significance of single-leg movement tasks compared with double-leg tasks with regard to valgus instability were described by [37], who reported that FPPA was significantly greater in the SLS (single-leg squat) than in the DJ (drop jump) (*p* < 0.001). A literature review about the dynamic knee valgus in the variations in single-leg movement tasks and training options was presented by [20].

In all jump tests (Tests 7–10), the analysis algorithm checks for short changes in the acceleration of the tibia and measures time intervals between certain events. These events are defined by heuristically specified constraints, or fixed or relative thresholds, for example, on the rising edge of the acceleration curve when landing after a jump, which perhaps could differ due to changed motion behavior. It remains to be tested whether the pattern recognition would still work correctly for all kinds of patients and changed test conditions.

When comparing the results with literature values, it must be considered that other investigators may define parameters differently. For example, in Test 7 Drop Jump, the IMU system integrates the medial acceleration only across the first maximum, i.e., up to the maximum medial speed of the knee joint, not up to the maximum medial shift of the tibia as one might expect.

Different definitions, for example, of the coordinate system or physiological axis, plane and angles, are not uncommon in biomechanics [38] and reflect the complex structure of the human musculoskeletal system, which cannot easily be reduced to a simple model. In order to obtain a certain degree of comparability, one should follow the general reporting standards of the International Society of Biomechanics (ISB) [39]. (This problem could become even more apparent in the future if neural networks derive the definition of score parameters and evaluation criteria of increasing abstractness on their own from the sensor measurements in a large database but with poor explanatory ability [40].)

Over time, some users would probably like to learn more about the movement characteristics of the patients performing these tests, especially with the more complex multi-component movement patterns of Tests 5–7, perhaps as an option of an “expert-user-mode” of IMU system software with the output of more complex information. If somehow feasible, the storage of the entire original sensor data set would be desirable for (optional) later post-processing and for a complete documentation, i.e., a step towards a measuring system in the literal sense. In the guidelines to IMU selection detailed in [41], sufficient on-board storage and the recording of the complete data are seen as prerequisites for use in pervasive healthcare-related studies and gait analyses. The possibility of installing further or improved algorithms with the help of system software updates, thus re-evaluating past measurements if necessary, is actually standard for electronic devices.

## 5. Conclusions

The decisive results for the physiotherapist can be summarized as follows: The angle ROM obtained in Tests 1–4 and Tests 8–10 (jump tests) can be assumed to be valid. There is no reason to think that the system would measure incorrectly when used with clinical patients, as long as the tests can be performed as described. The score differences between the two systems were within 1–3 degrees for angle measurements and almost identical for the timing-related jump tests. However, the validation of the valgus (in-)stability tests of the knee, especially of the two-legged drop jump test, can only be finally completed by comparable validation measurements on patients with actual instabilities. At this point, the user of the IMU system should not blindly rely on the results obtained until a study could also clearly validate these tests, that is, with patients as subjects.

The studied IMU system could bring a real improvement in the objective evaluation and documentation of treatment progress after lower-limb injuries in rehab or physiotherapy practice. The decisive advantage of the system is its simple and fast application, which does not interfere with the normal processes in practice.

A very practical feature of the system for the therapist is the provision of a test set from which the most suitable tests can be compiled with regard to the cause of the disease and the current state of therapy. No setup changes or calibrations are necessary.

The implemented tests are established in rehabilitation and physiotherapy and in the majority are validated by studies. In addition to the therapeutic benefit, some of them even have evidenced medical diagnostic value [17,21,22,25,26].

## 6. Limitations

The number of subjects (n = 5) in this study was low due to the large time required for the video-MA. A more important reason for that, however, was the foreseeable limited significance of three tests with the expected score values close to zero in the healthy subjects analyzed. The extrapolation to the expected results in patients, for example, with greater valgus instability of the knee, was, therefore, not possible.

A repetition of the three tests, especially of the drop jump test, with clinical patients is planned, but not before a thorough reconsideration of the associated evaluation algorithms.

## Figures and Tables

**Table 1:** Measured test scores of IMU system (IMU) and video-MA (VMA). All numerical values are averages over all volunteers and all test repetitions. SD (standard deviation) and RMS (root mean square) are variations of the score value differences between IMU and VMA. Percentage SD (%) and RMS (%) are differences relative to the VMA score.

Tests			Measured Score Values			Comparison of IMU and VMA Results			
No.	Set	Motion Pattern	Unit	IMU Score	VMA Score	SD	RMS	SD (%)	RMS (%)
1	A	Knee flexion (active)	(degrees)	97.5	96.5	6.03	6.12	6.2	7.4
2	A	Ankle extension	(degrees)	37.3	36.0	1.62	2.05	4.5	5.8
3	A	Knee flexion (passive)	(degrees)	127.6	124.6	3.36	4.51	2.7	3.4
4	A	Knee extension (passive)	(degrees)	25.5	24.9	1.2	1.34	4.8	5.7
5	B	One-leg stance (stability)	(mm)	2.5	2.7	0.4	0.41	14.8	19.3
6	B	One-leg squat (knee valgus)	(degrees)	3.78	3.77	1.23	1.35	32.6	53.9
7	B	Drop jump (knee valgus)	(degrees)	1.29	2.57	1.77	2.19	68.9	99
8	C	Vertical jump height	(cm)	20.5	20.5	0.77	0.77	3.8	3.8
9	C	Side hop	(counts)	52.2	52.2	-	-	-	-
10	C	Speedy jump	(s)	7.84	7.71	0.12	0.18	1.6	2.3

## Data Availability

The data presented in this study are available upon request to the corresponding author.
